# Intense Pulsed Light Therapy with Optimal Pulse Technology as an Adjunct Therapy for Moderate to Severe Blepharitis-Associated Keratoconjunctivitis

**DOI:** 10.1155/2019/3143469

**Published:** 2019-09-16

**Authors:** Fang Ruan, Yunxiao Zang, Ruti Sella, Hongshuang Lu, Shang Li, Ke Yang, Tao Jin, Natalie A. Afshari, Zhiqiang Pan, Ying Jie

**Affiliations:** ^1^Beijing Institute of Ophthalmology, Beijing Tongren Eye Center, Beijing Tongren Hospital, Capital Medical University, Beijing Key Laboratory of Ophthalmology and Visual Sciences, Beijing, China; ^2^Department of Ophthalmology, Beijing You'an Hospital, Capital Medical University, Beijing, China; ^3^Shiley Eye Institute, Department of Ophthalmology, University of California San Diego, La Jolla, San Diego, CA, USA

## Abstract

**Purpose:**

To evaluate the intense pulsed light (IPL) therapy with optimal pulse technology (OPT, M22™, Lumenis, USA) as an adjunct therapy for the prevention of recurrences in moderate to severe blepharokeratoconjunctivitis (BKC).

**Methods:**

This open-label nonrandomized clinical trial evaluated 33 patients diagnosed with BKC. Twenty-one patients received four bilateral OPT therapy sessions with Meibomian gland expression (MGX) (treatment group), and 11 patients received MGX alone (controls). This trial was initiated after a four-week pharmacotherapy for BKC in both groups and was scheduled at four-week intervals. Efficacy outcome measures included meibum quality, Meibomian gland (MG) secretion function, eyelid margin signs, corneal fluorescein staining (CFS) score, noninvasive keratography breakup time (NIKBUT), ocular surface disease index (OSDI) score, Schirmer I test (SIT), classification of tear film lipid layer (TFLL), and Meibomian gland dropout (MGDR). Safety outcome measures included visual acuity, intraocular pressure, eye structure damage, and facial skin appearance at each visit.

**Results:**

Quality of meibum, MG expressibility, eyelid margin signs, and OSDI score showed a statistically significant greater improvement in the treatment group after one to three treatment sessions, compared to controls (*p* < 0.05). While these improved in both groups in comparison to baseline, the NIKBUT and upper and lower eyelid MGDRs significantly improved only in the treatment group (*p* < 0.05). No adverse events occurred in both groups. No BKC recurrences were noted in the treatment group.

**Conclusions:**

IPL is a safe and effective adjuvant treatment for BKC and possibly more effective in reducing eyelid margin inflammation and prevents recurrences than MGX alone. This trial is registered with ChiCTR-ONN-17013864.

## 1. Introduction

Blepharitis is a common subacute or chronic inflammation affecting bilateral eyelid margins' skin and mucosa, eyelash follicles, and other adnexal glands. When this chronic inflammatory disease of the palpebral margin is complicated with secondary conjunctivitis and keratopathy, it is clinically referred to as blepharokeratoconjunctivitis (BKC) [1]. The clinical manifestation varies, and the disease may be complicated with corneal infiltration, ulceration, and eventually scarring with a consequent loss of vision [2]. The severity of blepharitis-associated keratoconjunctivitis can be classified as mild, moderate, or severe and is thought to correlate to the severity of Meibomitis in these patients [3]. In addition to the conventional prescribed eye drops [4], which often include artificial tears, nonsteroidal anti-inflammatory drugs (NSAIDs), corticosteroids, and antibiotics, physical therapy, namely, hot compresses, eyelid massage, and eyelid cleaning, is often incorporated into the BKC treatment plan to reduce recurrences. Nevertheless, the effect is limited, and the disease is likely to relapse. Intense pulsed light (IPL, Quantum™, Lumenis, USA), as a technology of physical therapy, has been widely applied as part of the treatment of hirsutism, as well as chronic skin damage secondary to dermal vascular diseases or facial skin sun exposure [5, 6]. First reports of a relief in patients' acne rosacea symptoms following IPL therapy were published in the early 2000s [5, 7–9]. As physicians noticed a consequent improvement of their patients' dry eye symptoms, they started assessing the theoretical mechanisms of IPL treatment for MGD and concluded that the treatment may cause selective photothermolysis and reduction of bacteria and/or parasitic growth and provide a temporary local warming effect [10, 11]. Nowadays, IPL is an emerging treatment option for patients with evaporative dry eye disease.

Since BKC and MGD share some common pathological mechanisms, including inflammation, occlusion of Meibomian gland, and new blood vessels sprouting at the eyelid margin, we hypothesized that the IPL may also carry an anti-inflammatory effect in BKC patients. Compared with the original IPL technology, the fifth generation of IPL with optimal pulse technology (OPT, M22™, Lumenis, USA) has better safety, efficacy, and reproducibility that can eliminate energy peak at the beginning of the pulse, avoid ineffective decline at the end of the pulse, and provide homogeneous “squared off ” energy distribution with continuous contact cooling [12]. We therefore aimed at evaluating the OPT as an adjunct blepharitis treatment for the prevention of recurrences in patients with previous active BKC.

## 2. Materials and Methods

### 2.1. Study Design

We conducted an open-label nonrandomized controlled clinical trial, which enrolled moderate to severe BKC adult patients from January 2018 to February 2018 in the Ophthalmology department of the Beijing Tongren Hospital, Capital Medical University. This study was approved by the ethics committee of the Beijing Tongren Hospital, Capital Medical University, and all participants had signed informed consents before treatment was initiated. All the examination procedures were done in accordance with the Declaration of Helsinki and ethics standards as well as specifications of the Chinese clinical trial research studies. The study was enlisted in the clinical trial registry (trial registration no.: ChiCTR17013864).

In all recruited patients, the keratoconjunctivitis was first controlled with topical eye drops for one month, and then the OPT combined with Meibomian gland expression (MGX) (treatment group) or MGX therapy alone (controls) were individually suggested. Treatment was initiated by the patient's preference and was repeated at monthly intervals for four consecutive months. The baseline and the four follow-up visits were coded as *V*0, *V*1, *V*2, *V*3, and *V*4. Subjective symptoms and objective signs were examined and recorded by a single cornea specialist (Y.J.) at each visit.

### 2.2. Enrollment Criteria

The patients in this study met all of the following inclusion criteria: (1) age older than eighteen; (2) bilateral disease; (3) documented signs of blepharitis, including eyelid hyperemia, capillary dilation, scales, scabs, ulcers of the eyelash root, and/or morphological changes of the Meibomian glands; (4) having concomitant conjunctival and corneal lesions, namely, conjunctival congestion, papillary hyperplasia (papillary tarsal conjunctival inflammation) [3], follicular formation or blister conjunctivitis, corneal peripheral punctate epithelial erosions, infiltration or ulceration, and/or corneal opacity with neovascularization.

### 2.3. Exclusion Criteria

Patients were excluded from the study if their diagnosis was not consistent with blepharitis-associated keratoconjunctivitis or if they had any of the following conditions: (1) acute inflammation or allergic eye or periocular skin disease; (2) underlying diseases that can be triggered by an exposure to wave lengths between 560 nm and 1200 nm, such as recurrent herpes simplex infections, systemic lupus erythematosus, or porphyria; (3) current pregnancy or lactation; (4) history of radiotherapy or chemotherapy treatment within the first year prior to the study or scheduled radiotherapy or chemotherapy within the two months after the planned OPT treatment.

### 2.4. Treatment

#### 2.4.1. Drug Therapy

Initial treatment was decided upon the degree of corneal and conjunctival pathology. One drop of 0.1% fluorometholone (5 ml : 5 mg, FML, Allergan, USA) was topically administered three or four times a day; one drop of 0.3% gatifloxacin eye gel (5 g, DIYOU, Shenyang Xingqi, China) was topically administered once or twice daily; and one drop of 0.3% sodium hyaluronate artificial tears (5 ml : 15 mg, AILI, Santen, Japan) was administered four times a day. The keratitis was reexamined after two weeks of treatment. The dosage of corticosteroid eye drops was then gradually tapered, and the other eye drops were discontinued in all the patients within one month of the resolution of corneal manifestations. Only sodium hyaluronate eye drops were continued two to three times per day thereafter. For patients with facial seborrheic dermatitis or acne rosacea, a dermatologist was consulted to determine the appropriate systemic drug regimen. Patients with rosacea were treated with minocycline hydrochloride capsules 50 mg (Wyeth Pharmaceuticals, China), twice a day, and with Fusidic cream (5 g : 0.1 g, Aomei Pharmaceutical, China) twice a day. Patients with seborrheic dermatitis were given oral dantone capsules 0.25 g, three times a day (Hili Pharmaceuticals, China), and selenium disulfide lotion (2.5%, Disano, China) was used twice a week. Oral medications were stopped before OPT + MGX or MGX was initiated.

#### 2.4.2. OPT/MGX versus MGX Treatment

After one month of topical drug treatment, keratoconjunctivitis was resolved in all subjects and the treatment of OPT/MGX or MGX alone was initiated in the treatment and control groups, respectively. All treatment sessions were performed by a single physician (Y.J.) using M22 OPT technology of Lumenis Medical Laser Co., Ltd. The procedure was performed taking the following measures:

*(1) Eye Protection*. Wet dressings were applied for skin and periocular hair protection. The physician was instructed to wear protective glasses.

*(2) Intensity Adjustment*. An 8 mm × 15 mm optical crystal cooling head was used, and the treatment parameters were tailored upon the patients' skin types [13]. Based on this previous study, for Fitzpatrick skin type III, the recommended settings were energy density 14 J/cm^2^, optical filter 560 nm, pulse quantity 3, pulse time 3.5 ms, and pulse delay 20 ms. For Fitzpatrick skin type IV, the settings were energy density 12 J/cm^2^, optical filter 590 nm, pulse quantity 3, pulse time 3.5 ms, and pulse delay 25 ms. Both cheeks were exposed to a test flare, and in the lack of any skin reaction, treatment was initiated 5 minutes later.

**Figure 1:**
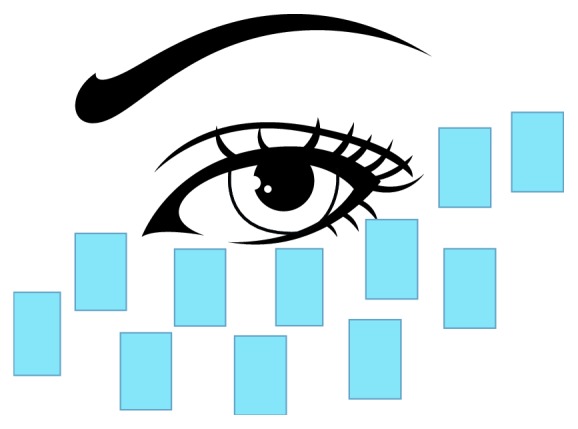
Treatment area by OPT.

*(3) OPT*. Medical ultrasonic couplant (250 g, Jinnuote, China) was applied to locate the treatment areas. Eleven points were then marked, including eight points at the lower eyelid margin in two lines from medial to lateral, two more points at the outer canthus, and one more point at the nasal alar. The treatment was done symmetrically on both sides. The coupling gel layer covering the treatment area was about 1-2 mm in thickness. The upper eyelid was not treated directly to avoid a possible light damage to the intraocular structures. The optical crystal directly touched the coupling gel in this area and lightly touched the skin, avoiding any pressure exertion. A pulse was emitted every 1-2 seconds. The coupling agent was removed after two repeated therapy sessions in the treatment area (Figure 1).

*(4) MGX*. A single 0.5% proparacaine hydrochloride eye drop (15 ml/75 mg, Alcaine, Alcon, Belgium) was applied to the conjunctival sac. Then, the Yoshitomi Meibomian Gland Compressor (AE-4521, ASICO, USA) was used to perform the Meibomian gland massage on the upper and lower eyelids. A small amount of tobramycin dexamethasone eye ointment (3.5 g, TobraDex, Alcon, Belgium) was administered to the palpebral margins after the double eyelids' massage.

*(5) Treatment regimen*. OPT/MGX or MGX therapy alone was initiated one month after pharmacotherapy was first started and was repeated four times at one-month intervals. Four follow-up visits were scheduled at week 4 (*V*1), week 8 (*V*2), week 12 (*V*3), and week 16 (*V*4) after the first therapy session to assess the eyelid margin, cornea, and conjunctiva by the same ophthalmologist.

### 2.5. Follow-Up

All the patients had their baseline eye exam recorded before their initial treatment with OPT/MGX or MGX alone. The examinations were performed at the following sequence: visual acuity, noncontact IOP (TX20, Canon, Japan), slit-lamp examination, and direct ophthalmoscopy examination were performed first, followed by the BKC-related examinations 30 minutes later. These included CFS score, NIKBUT, OSDI, SIT grading, grading of TFLL, and MGDR. The sequence of examinations was kept identical for each follow-up visit, and they were performed by the same single ophthalmologist (F.R).

Primary outcome measures of treatment's efficacy included quality of meibum, the expressibility of the Meibomian glands, and changes of eyelid margin. Secondary outcome measures of efficacy were assessed by CFS, NIKBUT, OSDI, SIT, grading of TFLL, and MGDR.

Outcome measures were assessed as follows.

#### 2.5.1. Quality of Meibum

Meibomian gland evaluator 1000 (MGE, Tear Science, USA) was located 1-2 mm inferior to the eyelid margin, and the central eight glands of the upper and lower eyelids were gently pressed. The liquid extracted from the Meibomian glands was graded by a classification method described by Bron et al., by which 0 = clear fluid, 1 = cloudy fluid, 2 = cloudy particulate fluid, and 3 = inspissated, toothpaste-like discharge. Each of the central eight glands was separately graded, and a score ranging from 0 to 24 was then given to each eye [14].

#### 2.5.2. Expressibility of the Meibomian Glands

The central five glands of the upper and lower eyelids were pressed. The following grading system based on Pflugfelder et al. study was used, by which 0 = all glands were expressible, 1 = 3-4 glands were expressible, 2 = 1-2 glands were expressible, and 3 = no glands were expressible. Score ranged from 0 to 3 points [15].

#### 2.5.3. Changes of Eyelid Margin

Evaluation of the eyelid margin status was based on the following five signs: blunt rounding shape of the posterior eyelid margin, irregularity or notching of the eyelid margin, and the presence of trichiasis or distichiasis, anterior blepharitis, vascularity, or telangiectasia of the lid margin. One point was assigned to each clinical sign, and the grade ranged from 0 to 5 points [14].

#### 2.5.4. CFS Score

Using the Fluo Imaging corneal dot stain observation program (K5M, Oculus Keratograph 5M, Oculus Optikgerate GmbH, Germany), the cornea was divided into five regions and graded based on dye distribution on the background of cobalt blue light. Grading ranged from 0 to 15 as follows: 0 = no staining; 1 = 1–5 punctate staining; 2 = 6–15 punctate staining; and 3 = any of the following: ≥16 punctate staining, ≥1 long 1 mm staining sites, any filamentous staining [16].

#### 2.5.5. NIKBUT

Using the K5M tf-scan tear film analysis procedure, the quality and the stability of the tear film were evaluated by a noncontact and fully automatic method. The device automatically recorded the time at first tear breakup point and its location, starting measurements 1.5 seconds after the patient's second blink. Values below 10 seconds were considered pathological.

#### 2.5.6. OSDI Score

The OSDI questionnaire was used to assess the extent of patients' discomfort. The questionnaire included 12 questions [17]. Final grade ranged from 0 to 100 points, and patients with 0–12 points were classified as asymptomatic, patients with 13–32 points were classified as mild to moderate, and patients with 33–100 were classified as severe.

#### 2.5.7. SIT

The Schirmer test I was performed using a filter paper (5 mm × 35 mm Whatmann no. 41) placed inside the lower eyelid. The filtered paper was taken out after five minutes, and the amount of wetting was measured in millimeters. Exam was considered positive if wetting of the paper was 5 mm or less.

#### 2.5.8. Grading of TFLL

Using the K5M, the thickness and stability of the lipid layer were evaluated and the TFLL score ranged from one to five as follows: grade 1: gray, uniform stripes; grade 2: grayish white, but with slight stripes change; grade 3: yellow stripes appear; grade 4: a jumble of colored streaks; and grade 5: black dry spots [18]. A grade >3 was considered abnormal.

#### 2.5.9. MGDR

Using the Meibo-Scan of the K5M, the structure of the Meibomian glands was observed by an infrared light source, and the loss of the glands was then scored from one to three as follows: 1 = the loss of Meibomian glands was less than 1/3 of the total area; grade 2 = the loss of Meibomian glands accounted for 1/3 to 2/3 of the total area; and grade 3 = the loss of Meibomian glands accounted for 2/3 or more of the total area [19].

### 2.6. Safety

The skin at the treatment site was evaluated for any temporary pigmentary changes, alterations in skin sensation, including tingling, itching, or burning, rashes or blisters, skin edema, signs of an active Herpes Simplex virus infection, or inflammatory hypertension. The ETDRS best-corrected visual acuity and the intraocular pressure were measured before and after treatment. slit-lamp biomicroscopy was performed to rule out any conjunctival, corneal, iridal, or lenticular damage. Any iris depigmentation was documented. The direct ophthalmoscope was then used to perform a dilated fundus exam.

### 2.7. Statistical Methods

SPSS 19.0 (IBM, USA) was used for statistical analysis. One-way repeated measure analysis of variance enabled comparison of data across the various time points, paired analyses allowed comparison of pre- and posttreatment data at individual time points and multifactor variance analysis permitted comparison between the two groups. Data are reported as mean ± SD. *p* value ≤ 0.05 was considered statistically significant.

## 3. Results

Among the 21 adult BKC patients (42 eyes) who consisted the treatment group, there were 13 women (61.9%) and 8 men (38.1%) with a mean age of 42.93 ± 13.25. The 11 adult BKC patients (22 eyes) in the control group consisted of seven women (63.6%) and four men (36.4%) with a mean age of 47.62 ± 14.92.

### 3.1. Primary Outcome Measures

**Figure 2:**
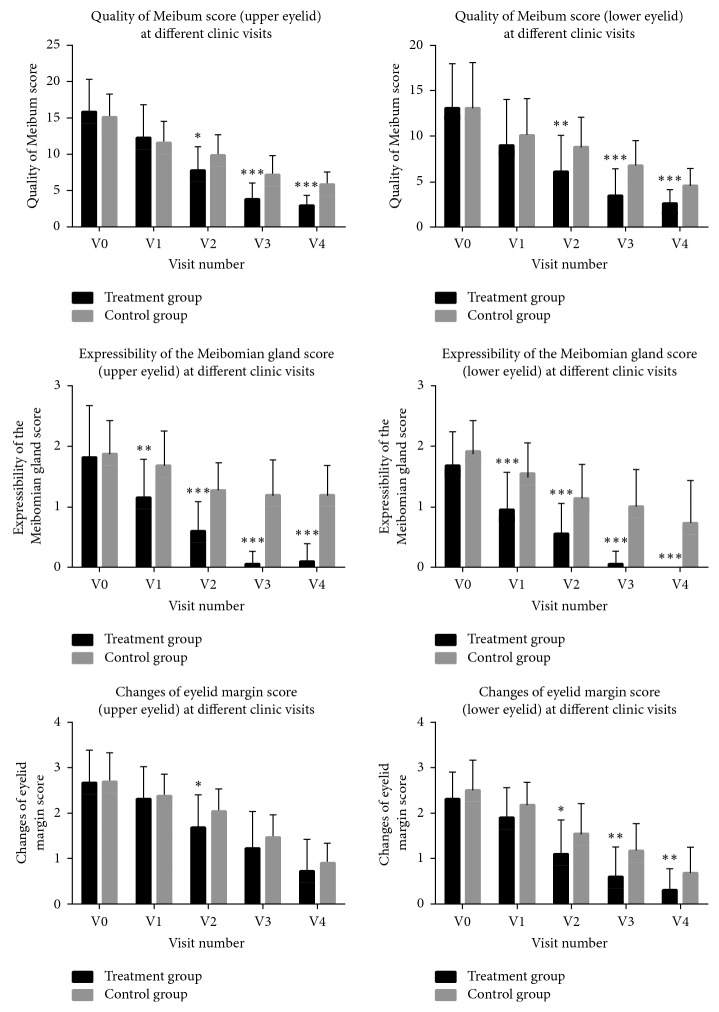
Comparison of quality of meibum secretion, expressibility of the Meibomian glands, and changes of eyelid margin of upper and lower eyelids between the treatment and control groups at different follow-up time points. The baseline and the four follow-up visits were coded as V0, V1, V2, V3, and V4. ^*∗*^*p* < 0.05, ^*∗∗*^*p* < 0.01, and ^*∗∗∗*^*p* < 0.001.

As shown in Table 1 and Figure 2, the quality of meibum excretion and MG expressibility significantly improved in both the treatment group and the controls with more treatment sessions (OPT/MGX or MGX) applied, and improvement was greater in the treatment group when comparing the Meibum quality of both the upper and lower eyelids (*p*=0.014 and 0.008, respectively) from the second treatment session and on, while the difference in MG expressibility became evident as early as the first session (*p*=0.002 and <0.001, respectively). Differences remained significant for the entire follow-up period.

Eyelid margin signs improved in both groups of patients. A statistically significant difference between the groups was evident in the lower eyelids, as soon as the second visit (*p*=0.022), and kept significant till the last visit, while in the upper eyelids, a statistically significant difference was noted only at the second visit (*p*=0.041).

### 3.2. Secondary Outcome Measures

**Figure 3:**
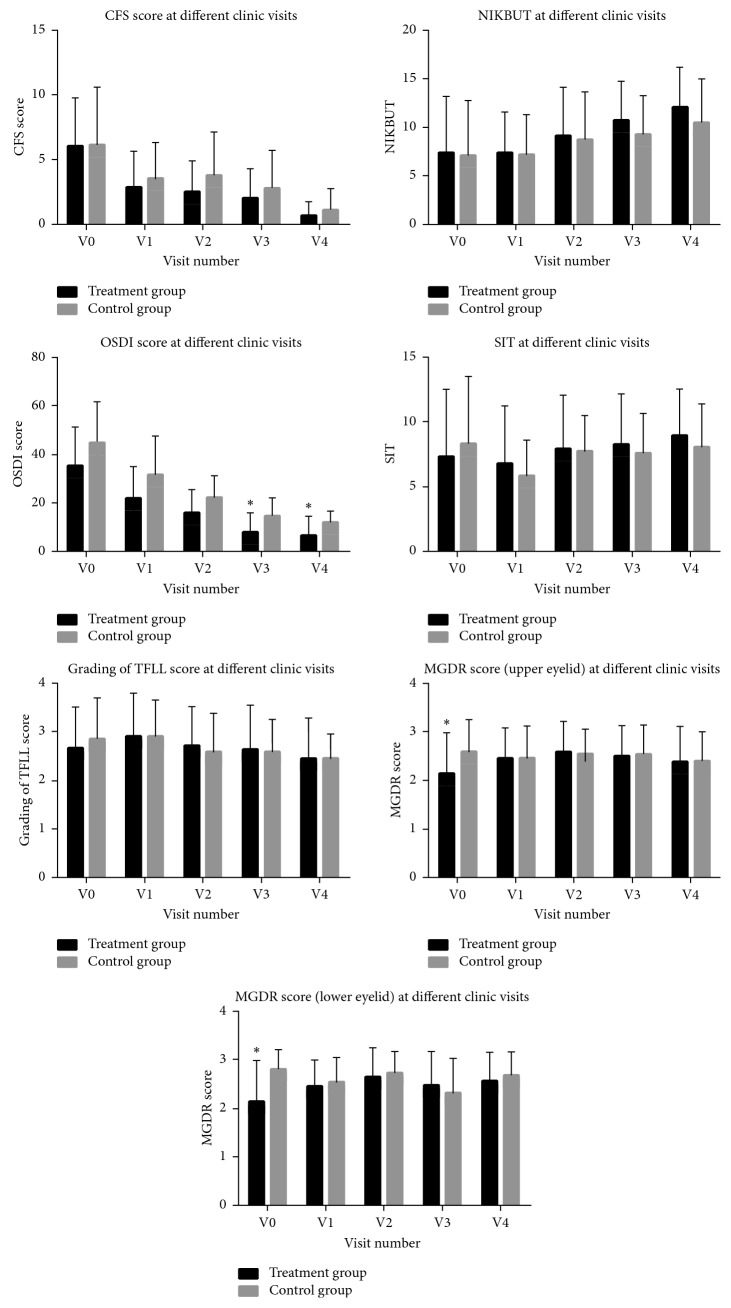
Comparison of CFS scores, NIKBUT, OSDI, SIT, classification of tear film lipid layer, and Meibomian gland dropout of upper and lower eyelid between the treatment and control groups at different follow-up time points. The baseline and the four follow-up visits were coded as *V*0, *V*1, *V*2, *V*3, and *V*4. ^*∗*^*p* < 0.05, ^*∗∗*^*p* < 0.01, and ^*∗∗∗*^*p* < 0.001.

As demonstrated in Table 2 and Figure 3, CFS scores and NIKBUT results were similar between the treatment and control groups (*p* > 0.5). Nevertheless, during the treatment sessions CFS scores significantly differed from baseline in both groups, while the difference in NIKBUT in comparison to baseline was only observed in the treatment group. Subjective symptoms, as reflected by the OSDI, significantly improved after the first therapy session in both groups, and the treatment group showed more subjective improvement as soon as the third follow-up visit and thereafter (*p*=0.029 at *V*3 and *p*=0.049 at *V*4). No statistically significant differences were observed among the two groups when comparing the SIT and tear film lipid layer classification, as seen in Table 2. Though the baseline MGDR was significantly lower in the treatment group in both upper and lower eyelids, an improvement throughout the follow-up period occurred solely in this group (*p*=0.044 for upper eyelid and *p*=0.016 for lower eyelid).

Five patients in the treatment group (23.8%) and three patients in the control group (27.3%) had associated dermatologic diseases. After four OPT sessions, the ocular signs and facial skin lesions showed much improvement, indicating high patient satisfaction, while there was no change in dermatosis in the control group.

### 3.3. Adverse Effects

None of the participants in the treatment group experienced a decrease in best-corrected visual acuity, and intraocular pressures were measured <21 mmHg in all eyes. Only three of the patients (14.3%) reported a burning sensation at the area treated with OPT, but the symptoms resolved after the adjustment of energy parameters without further influencing the treatment. Only 1/21 patients endured hair loss attributable to the proximity of the treatment area to patient's hair line; however, hair regrowth soon pursued on the next visit. No recurrence of BKC was observed in the treatment group, while 2/11 patients (18.2%) in the control group had a documented BKC recurrence at the second or third visit, respectively. No other adverse effects were documented.

## 4. Discussion

Blepharokeratoconjunctivitis represents a group of recurrent corneal and conjunctival diseases associated with anterior and posterior blepharitis [2]. Posterior blepharitis is thought to play a more significant role in the occurrence of BKC; hence, modification of its name to Meibomitis-related keratoconjunctivitis (MRKC) has been previously suggested [20]. Misdiagnosis of BKC is not uncommon given the subtle nature of the eyelid margin signs and the irreversible tissue damage with a possible consequent vision impairment which may follow [21].

The beneficial effect of IPL as a treatment modality for MGD has been previously reported, with significant improvement in TFLL, BUT, subjective symptom scores, and eyelid margin signs, especially for those with refractory MGD [22–26]. The pivotal mechanism behind IPL with OPT is the induction of selective photothermolysis of oxyhemoglobin of the yellow light, transforming luminous energy into heat energy, enabling coagulation and ablation of abnormal capillaries which also decreases the dissemination of inflammatory factors [6, 27]. This is seen in its effect over various diseases, including rosacea [5]. It is also utilized for the reduction of *Demodex folliculorum* mites and *Bacillus oleronius* bacterium which are potential mediators of blepharitis and MGD [28, 29], and it has a temporary local thermal effect which can melt meibum to facilitate its secretion.

In this study, we show that the IPL of Lumenis M22 with OPT, as an adjunctive therapy to MGX, is a viable therapy for BKC patients and may prevent keratoconjunctivitis recurrence by controlling blepharitis. The IPL treatment in our study patients was initiated immediately after the completion of a one-month topical steroidal treatment, and a clinical assessment that active inflammation has resolved. Though the lasting anti-inflammatory effect of the eye drops could have potentially played a role in the late improvement of eyelid signs, both patients and controls have completed the same drop regimen.

Our results show that the OPT can significantly and effectively ameliorate the quality of meibum, improve MG expressibility, and regress eyelid margin signs and subjective symptoms in BKC patients more effectively than the traditional MGX therapy. As previously shown [3], reducing the inflammation of the eyelids by promoting the expression of Meibomian gland secretions and improving the quality of meibum are important steps for the treatment of BKC and for the prevention of its recurrence. Moreover, there is a very good correlation between the Meibomitis and the corneal and conjunctival signs in this group of patients, as previously described by Suzuki et al.

Interestingly, the OPT treatment achieved more improvement of lower eyelid signs from the second visit and thereafter, while the effect of treatment on the upper eyelids signs was more modest. One optional explanation for this discrepancy is the application of IPL at the cheek region, which lies in greater proximity to the inferior palpebral margin. A recent study by Rong et al. [11] reported that in patients receiving IPL treatment on both the upper and lower eyelids, a significant difference in the quality of meibum of the upper eyelid in comparison with the control group was already noted on day 28 after the first treatment session, while significant differences in our study appeared at the second visit (day 56). We therefore suggest it may be advisable to apply OPT to both the upper and lower eyelids with a proper eyeball protection to gain a better therapeutic effect. Notably, our study was not the first to have demonstrated a beneficial effect over the upper eyelids with IPL treatment limited to the cheek area [30]. While the mechanism for this indirect effect is not entirely clear, we presume that the decrease in secretion of proinflammatory agents from abnormal blood vessels which are affected by the treatment has a local and regional impact on both eyelids. Our results also indicate an early response to the IPL treatment as represented by MG expressibility. This finding can be supported by the previously suggested tear gradient theory, proposed by Bron et al. [31], linking the damage to MG orifices and the subsequent MGD, with the concentration of proinflammatory proteins in the tear meniscus. Since IPL can decrease inflammation in MGD patients, as previously described by Liu et al. [32], it is likely to be represented by an improvement in MG expressibility.

Our study shows that both the treatment and the control groups experienced an improvement in CFS scores, with no significant difference between the groups. This result could be related to the ongoing MGX maintenance treatment that both groups received, which is the traditional therapy for BKC, as previously reported by Rong et al. [11]. Corneal epithelial healing may therefore ensue once the MG function improves and may not be represented as a direct consequence of OPT treatment. The differences in NIKBUT between the treatment and the control groups were not significant in our study, as opposed to Rong et al. [11] and Craig et al. [23] who found significant differences in NIKBUT at days 28 and 45 after the first treatment session, respectively. One possible explanation is the wide spectrum of BKC and MGD severity in different studies. A study by Yin and Gong [33] found that Asian patients with BKC are characterized by a significant decrease in meibum quality and severe MG dropout. Hence, tear film stability may be a harder goal to achieve with treatment, given the challenging baseline gland status. The same could explain the lack of significant difference in classification of TFLL. In general, changes in the function of the glands, as manifested by their impact on the tear film content and stability, as well as cornea staining, may present at a later time point, in comparison to the rather early treatment impact on the Meibomian glands structure. These changes were, therefore, not established in our study, given the relatively short follow-up time. The comparison of MGDR between the groups was somewhat limited given the significant difference between the groups at baseline for both upper and lower eyelids. We could show, however, an improvement of MGDR in the treatment group during the follow-up time, as previously suggested by Yin et al. [34]. The authors suggested that IPL improves MG macrostructure, namely, MGDR, and MG microstructure (i.e., MG acinar longest diameter and MG acinar unit density) and decreases the inflammatory response in the MGs. Therefore, though the severity profile of MGD in BKC patients is usually worse than the average, IPL therapy may still be recommended and may be responsible for stimulating acinar cells and decreasing inflammation. In our study, the patients' subjective satisfaction measures, expressed by the OSDI scores, improved more in the IPL group, in concordance with previous studies [24, 35].

Since OPT-related uveitis and iris photoablation were previously described [36, 37], we enforced eye protection during the entire procedure. No uveitis episodes or adverse effect on vision were documented.

Our study is limited by its small sample size, its open-label nature, a relatively short follow-up, time and strict exclusion criteria of patients with comorbidities. Our preliminary results, however, indicate that IPL with OPT therapy may have an adjunctive effect to the conventional MGX in improving the function of Meibomian glands, controlling ocular surface inflammation, relieving ocular discomfort symptoms, increasing the stability of the tear film, preventing the recurrence of BKC, and avoiding the side effect of long-term drug use. It should therefore be considered an effective adjunct treatment for BKC, specifically in the presence inflammatory skin disorders.

## Figures and Tables

**Table 1:** Comparison of primary outcome measures.

Items	Groups	*V*0	*V*1	*V*2	*V*3	*V*4	*p* ^*∗*^
Quality of meibum (upper eyelid)	Treatment group	15.83 ± 4.43	12.17 ± 4.59	7.69 ± 3.35	3.79 ± 2.23	2.86 ± 1.44	**<0.001**
	Control group	15.05 ± 3.24	11.50 ± 2.99	9.82 ± 2.84	7.09 ± 2.71	5.77 ± 1.74	<**0.001**
	*p* ^#^	0.465	0.541	**0.014**	**<0.001**	**<0.001**	

Quality of meibum (lower eyelid)	Treatment group	13.07 ± 4.92	9.00 ± 5.08	6.17 ± 3.89	3.43 ± 2.96	2.55 ± 1.61	**<0.001**
	Control group	13.14 ± 4.97	10.14 ± 4.00	8.82 ± 3.29	6.77 ± 2.78	4.59 ± 1.87	<**0.001**
	*p* ^#^	0.960	0.366	**0.008**	**<0.001**	**<0.001**	

Expressibility of the Meibomian glands (upper eyelid)	Treatment group	1.62 ± 0.76	1.14 ± 0.65	0.60 ± 0.50	0.05 ± 0.22	0.10 ± 0.30	**<0.001**
	Control group	1.73 ± 0.53	1.68 ± 0.57	1.27 ± 0.45	1.18 ± 0.59	1.18 ± 0.50	**<0.001**
	*p* ^#^	0.052	**0.002**	**<0.001**	**<0.001**	**<0.001**	

Expressibility of the Meibomian glands (lower eyelid)	Treatment group	1.67 ± 0.57	0.95 ± 0.62	0.55 ± 0.50	0.05 ± 0.22	0.00 ± 0.00	**<0.001**
	Control group	1.91 ± 0.53	1.55 ± 0.51	1.14 ± 0.56	1.00 ± 0.62	0.73 ± 0.70	**<0.001**
	*p* ^#^	0.102	**<0.001**	**<0.001**	**<0.001**	**<0.001**	

Changes of eyelid margin (upper eyelid)	Treatment group	2.67 ± 0.72	2.31 ± 0.71	1.69 ± 0.71	1.21 ± 0.81	0.71 ± 0.71	**<0.001**
	Control group	2.68 ± 0.65	2.36 ± 0.49	2.05 ± 0.49	1.45 ± 0.51	0.91 ± 0.43	**<0.001**
	*p* ^#^	0.934	0.752	**0.041**	0.212	0.242	

Changes of eyelid margin (lower eyelid)	Treatment group	2.31 ± 0.60	1.90 ± 0.66	1.10 ± 0.76	0.60 ± 0.66	0.31 ± 0.47	**<0.001**
	Control group	2.50 ± 0.67	2.18 ± 0.50	1.55 ± 0.67	1.18 ± 0.59	0.68 ± 0.57	**<0.001**
	*p* ^#^	0.254	0.088	**0.022**	**0.001**	**0.007**	

^*∗*^ *p* value of one-way repeated measure analysis of variance to compare data for each group at different time points; ^#^*p* value of multivariate analysis to compare the treatment and control groups at a specific time point.

**Table 2:** Comparison of secondary outcome measures.

Items	Groups	*V*0	*V*1	*V*2	*V*3	*V*4	*p* ^*∗*^
CFS score	Treatment group	6.02 ± 3.74	2.86 ± 2.83	2.52 ± 2.38	2.05 ± 2.23	0.67 ± 1.07	**<0.001**
	Control group	6.14 ± 4.44	3.50 ± 2.82	3.82 ± 3.35	2.82 ± 2.92	1.14 ± 1.64	**<0.001**
	*p* ^#^	0.915	0.391	0.078	0.243	0.173	

NIKBUT	Treatment group	7.38 ± 5.79	7.44 ± 4.14	9.14 ± 4.97	10.71 ± 4.04	12.07 ± 4.14	**<0.001**
	Control group	7.10 ± 5.61	7.17 ± 4.16	8.73 ± 4.95	9.23 ± 4.06	10.53 ± 4.48	0.085
	*p* ^#^	0.857	0.803	0.759	0.168	0.175	

OSDI score	Treatment group	35.38 ± 15.76	22.08 ± 12.94	16.03 ± 9.54	8.18 ± 8.01	6.89 ± 7.61	**<0.001**
	Control group	44.76 ± 17.06	31.62 ± 15.89	22.32 ± 8.97	14.84 ± 7.32	12.05 ± 4.64	**<0.001**
	*p* ^#^	0.130	0.077	0.081	**0.029**	**0.049**	

SIT	Treatment group	7.31 ± 5.24	6.81 ± 4.49	7.93 ± 4.19	8.26 ± 3.91	8.93 ± 3.62	0.197
	Control group	8.36 ± 5.17	5.91 ± 2.71	7.77 ± 2.74	7.64 ± 3.05	8.09 ± 3.31	0.168
	*p* ^#^	0.445	0.393	0.875	0.516	0.370	

Classification of tear film lipid layer	Treatment group	2.67 ± 0.85	2.93 ± 0.87	2.71 ± 0.80	2.64 ± 0.91	2.45 ± 0.83	0.155
	Control group	2.86 ± 0.83	2.91 ± 0.75	2.59 ± 0.80	2.59 ± 0.67	2.45 ± 0.51	0.175
	*p* ^#^	0.377	0.929	0.561	0.813	0.991	

Meibomian gland dropout (upper eyelid)	Treatment group	2.14 ± 0.84	2.45 ± 0.63	2.60 ± 0.63	2.50 ± 0.63	2.38 ± 0.73	**0.044**
	Control group	2.59 ± 0.67	2.45 ± 0.67	2.55 ± 0.51	2.55 ± 0.60	2.41 ± 0.59	0.857
	*p* ^#^	**0.035**	0.990	0.750	0.782	0.877	

Meibomian gland dropout (lower eyelid)	Treatment group	2.17 ± 0.82	2.45 ± 0.55	2.64 ± 0.62	2.48 ± 0.71	2.57 ± 0.59	**0.016**
	Control group	2.82 ± 0.39	2.55 ± 0.51	2.73 ± 0.46	2.32 ± 0.72	2.68 ± 0.48	0.062
	*p* ^#^	**0.001**	0.512	0.574	0.401	0.452	

^*∗*^ *p* value of one-way repeated measure analysis of variance to compare data for each group at different time points; ^#^*p* value of multivariate analysis to compare the treatment and control groups at a specific time point.

## Data Availability

The data used to support the findings of this study are available from the corresponding author upon request.
